# Effectiveness of circular hexapod external fixation with soft tissue reconstruction in treating severe knee dislocation due to burn scarring: a case report

**DOI:** 10.1186/s12891-020-03667-7

**Published:** 2020-09-28

**Authors:** Koji Nozaka, Naohisa Miyakoshi, Hidetomo Saito, Hiroaki Kijima, Motoki Mita, Yoichi Shimada

**Affiliations:** Department of Orthopedic Surgery, Akita University Graduate School of Medicine, 1-1-1 Hondo, Akita, 010-8543 Japan

**Keywords:** Soft tissue reconstruction, Circular hexapod external fixation, Hinged total knee arthroplasty

## Abstract

**Background:**

Severe varus deformity and soft tissue injury caused by a burn around a complex knee dislocation is a rare injury. Soft tissue reconstruction and deformity correction with circular hexapod external fixation of the knee and hinged total knee arthroplasty (TKA) are challenging and can lead to major complications if not performed appropriately. We present a case in which a combination of soft tissue reconstruction, circular hexapod external fixation, and TKA was used treat severe knee dislocation due to burn scarring.

**Case presentation:**

We report the case of a 58-year-old woman who presented for knee surgery with soft tissue reconstruction, deformity correction with circular hexapod external fixation, and hinged TKA for a severe complex deformity and soft tissue injury caused by a burn injury at 6 months of age. The left leg was shorter by 35 mm in terms of functional leg length discrepancy. She walked with a limp, with a marked varus deformity of the left knee during the stance phase of walking. After a 3-stage repair, the patient was able to walk without assistance, confirming improvement of mobility.

**Conclusion:**

The treatment method was an effective use of a combination of soft tissue reconstruction, circular hexapod external fixation, and rehearsal surgery using a 3D printed bone model of the modular rotating hinge component of TKA, which was successfully used to treat a severe knee dislocation due to burn scarring. This staged surgery maintained the leg length and ultimately achieved a satisfactory alignment.

## Background

It is challenging to manage a severe complex varus deformity and soft tissue injury caused by a burn around the knee, years after the original injury. This would require several staged treatments with soft tissue reconstruction, deformity correction, and highly constrained or hinged implants. Previously, there have been several reports of arthrodesis with the Ilizarov device after failed knee arthroplasty [1–4]. Additional studies with a larger number of patients with severe injuries such as road traffic accidents, sports injuries, and war trauma around the knee, are needed to confirm the use of circular external fixation as a feasible and effective treatment option [5–8]. The use of circular hexapod external fixation to treat severe knee dislocation due to burn scarring has not been reported previously in the English literature. This method was adopted to overcome the difficulties and avoid the complications associated with common severe knee deformities with soft tissue disorder treatment. We present a case in which a combination of soft tissue reconstruction, circular hexapod external fixation, and TKA was used treat severe knee dislocation due to burn scarring.

## Case presentation

**Fig. 1:**
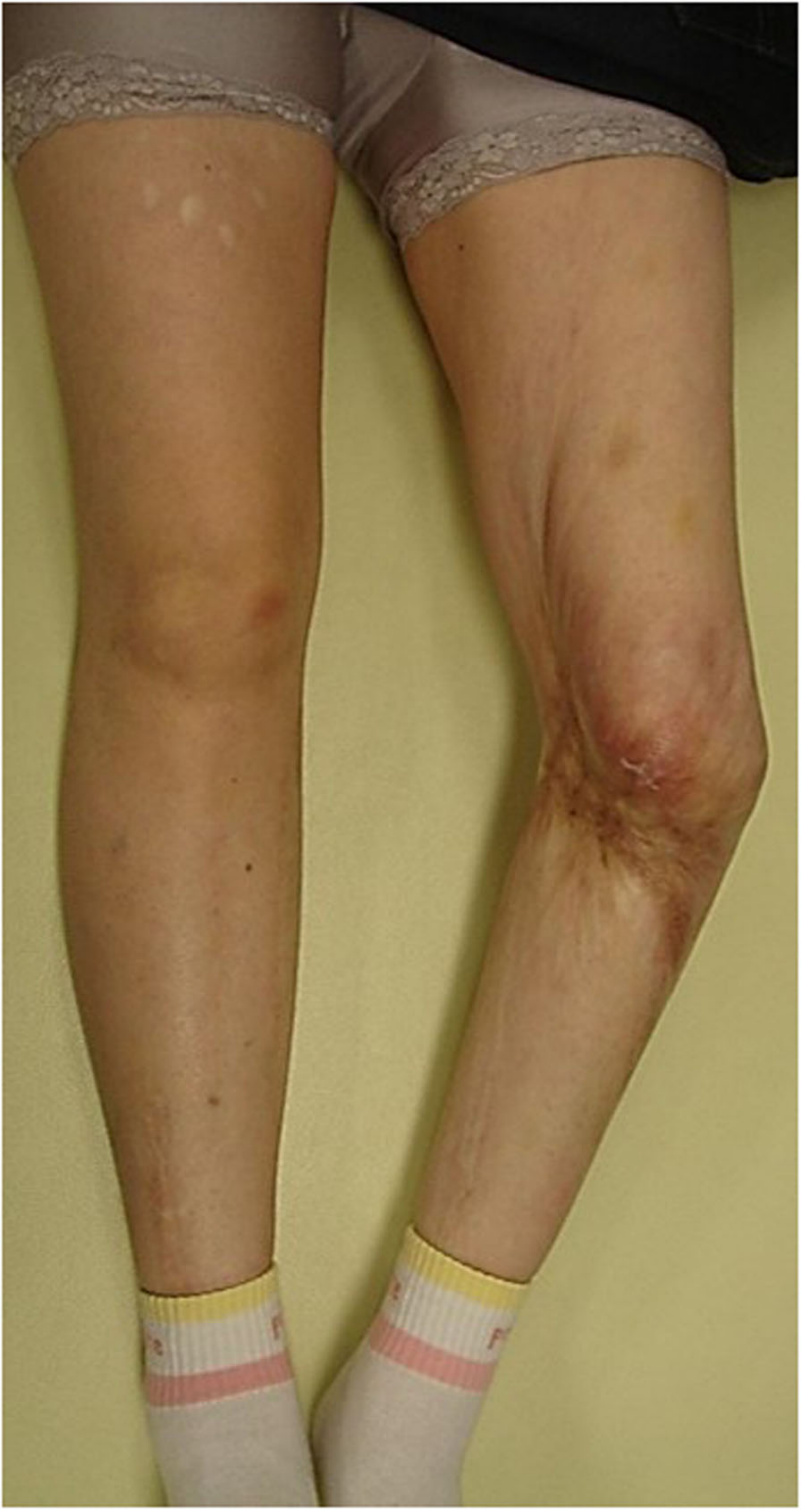
Large burn scar that extended from the proximal medial to distal anterior side of the left knee

**Fig. 2:**
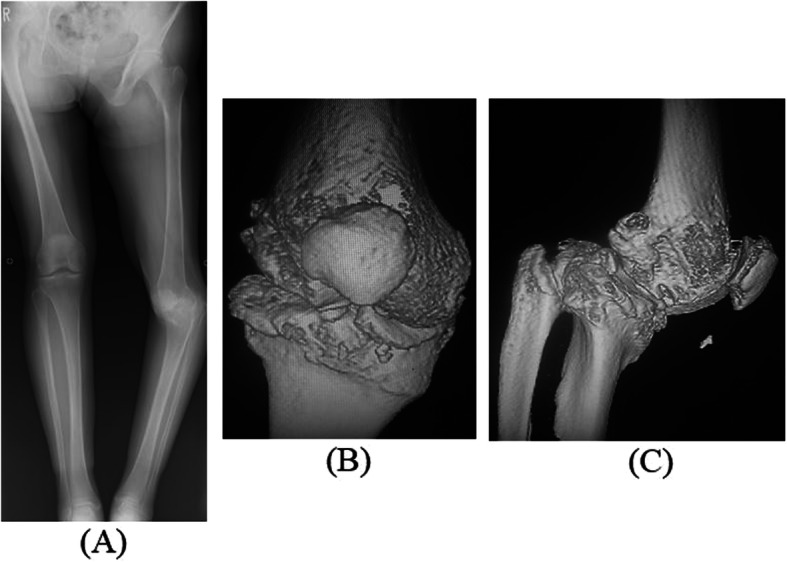
Preoperative X-ray and 3D CT. **a** Preoperative antero-posterior lower extremity full-length standing X-ray. **b** Preoperative anterior-posterior 3D CT. **c** Preoperative medial-lateral angle 3D CT

A 58-year-old woman complained of left knee pain. At 6 months of age, she had fallen behind the Japanese Irori fireplace in her house and sustained severe and extensive deep burns of the left lower extremity. Because of a wide burn scar on the left knee, during childhood, she developed a left knee contracture and severe varus deformity, internal torsion, and posterior knee dislocation. There was a large burn scar that extended from the proximal medial to distal anterior side of the left knee (Fig. 1). She walked with a limp and frequently fell because of leg length discrepancy secondary to the severe knee contracture and complex deformity. As she became an adult, the varus deformity and left knee pain worsened (Fig. 2). She was also a high-risk candidate for general anesthesia because of severe heart failure. In addition, her bone strength was low secondary to long-term non-weight bearing because of severe knee deformity and pain. Baseline dual-energy X-ray absorptiometry revealed that her left femoral neck bone mineral density was 0.30 g/cm^2^ before surgery.

**Fig. 3:**
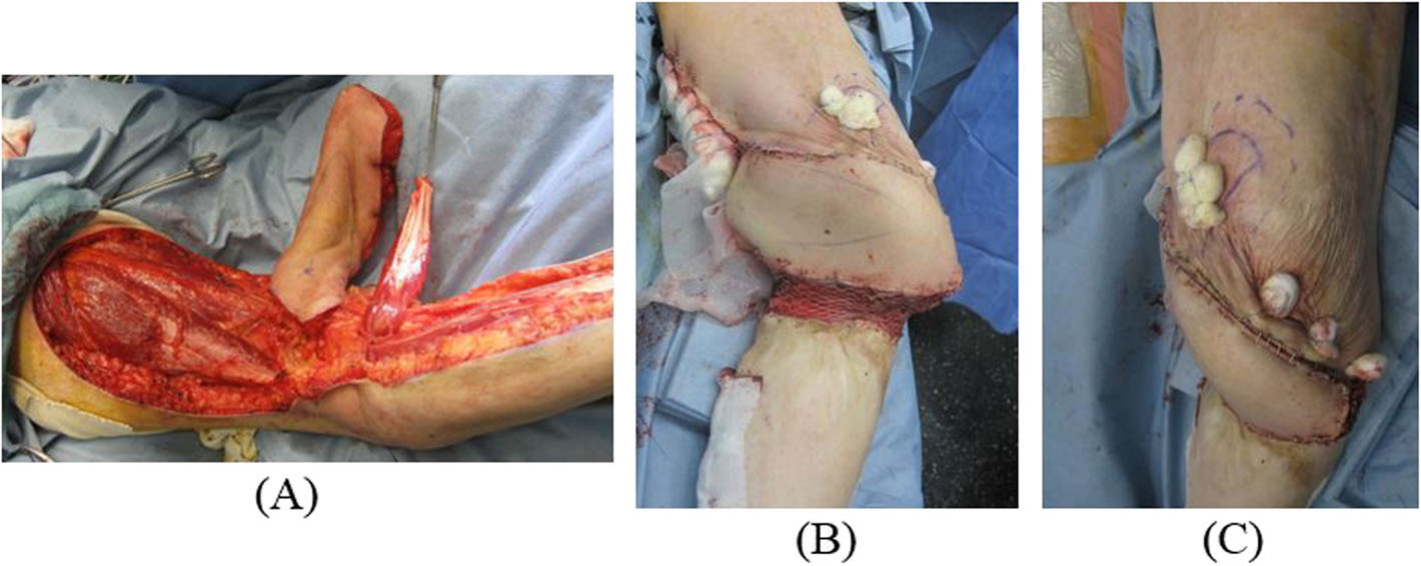
Soft tissue reconstruction with combined gastrocnemius flap and popliteo-posterior thigh fasciocutaneous island flap to treat the very wide burn scar. **a** The gastrocnemius flap and the popliteo-posterior thigh fasciocutaneous island flap in the prone position. **b** Immediate postoperative medial angle. **c** Immediate postoperative anterior angle

**Fig. 4:**
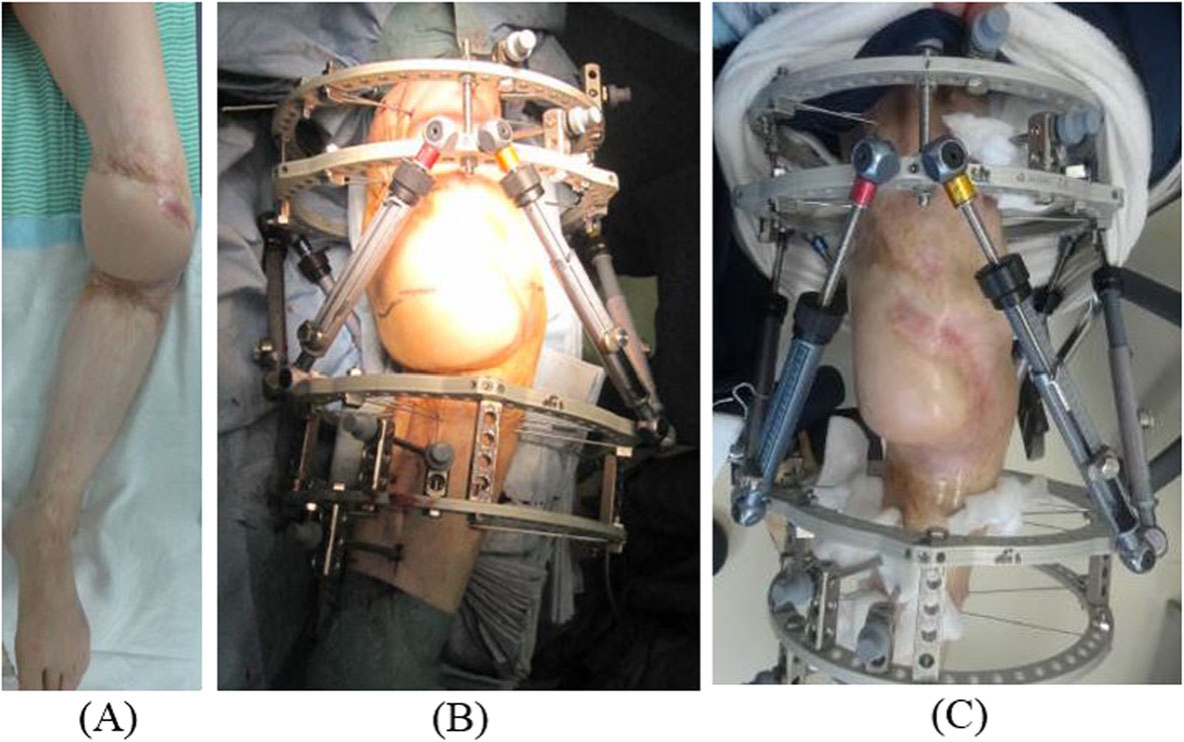
Clinical photograph. **a** The flap was engrafted 2 months after soft tissue reconstruction. **b** Immediate postoperative circular hexapod external fixation. **c** After correction deformity with circular hexapod external fixation

**Fig. 5:**
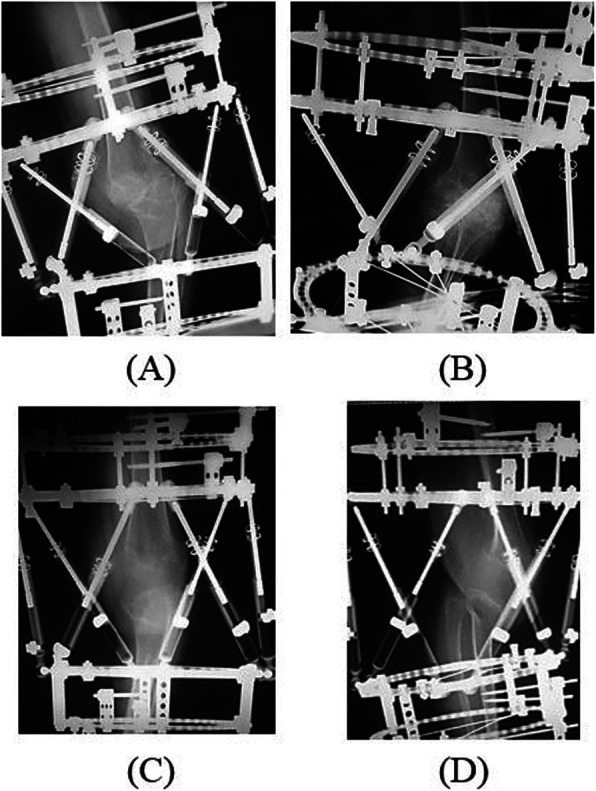
X-ray with circular hexapod external fixation. **a** Immediate postoperative antero-posterior X-ray with circular hexapod external fixation. **b** Immediate postoperative medio-lateral X-ray with circular hexapod external fixation. **c** Antero-posterior X-ray after correction deformity with circular hexapod external fixation. **d** Medio-lateral X-ray after correction deformity with circular hexapod external fixation

We initially performed soft tissue reconstruction with a combined gastrocnemius flap and popliteo-posterior thigh fasciocutaneous island flap to treat the very wide burn scar (Fig. 3) [9–13]. Eight weeks after reconstructive surgery of the soft tissue, we performed knee joint-bridging circular hexapod external fixation of the femur and the tibia [14–16]. Finally, gradual correction was carried out (Figs. 4, 5).

**Fig. 6:**
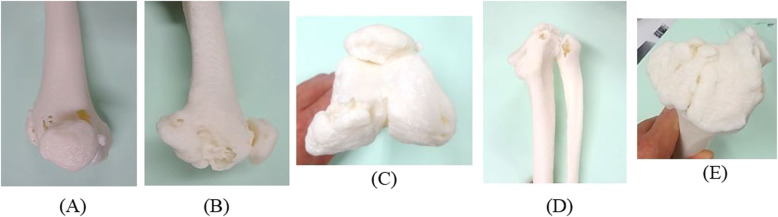
3D printed bone model. **a** Antero-posterior 3D printed bone model of femur. **b** Medio-lateral 3D printed bone model of femur. **c** Axial 3D printed bone model of femur. **d** Lateral-medial angle 3D of tibia. **e** Axial 3D printed bone model of tibia

Pre-surgical planning started from the quantification of the angular deformity, followed by computer simulated correction and, finally, a rehearsal surgery on 3D printed bone models for TKA (Fig. 6) [17, 18].

### Operative surgical procedure

#### 1st stage: soft tissue reconstruction

There was thin and atrophic soft tissue of a wide burn scar in the knee joint. We evaluated preoperative angiography and Doppler flowmeter results to evaluate the presence of the nutrient artery that may be used to guide the procedures safely [2]. We initially performed soft tissue reconstruction with musculocutaneous flaps of the medial head of the gastrocnemius muscle and the posterior aspect of the thigh. After creating the medial head of the gastrocnemius muscle flap, a leaf-shaped flap extended from the popliteal region to the gluteal sulcus. The incision was initially made parallel to the long axis and was carried down through the subcutaneous tissue and the deep fascia of the thigh. A distal incision was made and the distal end of the flap was gently turned upward. The islanded popliteo-posterior thigh fasciocutaneous flap was then elevated and transposed to the defect [9–13]. The flap was engrafted 2 months after soft tissue reconstruction.

#### 2nd stage: deformity correction

Alignment adjustment of TKA and gradual correction were carried out in advance, to avoid circulatory disturbance and neuropathy. A fluoroscopy machine was positioned on the contralateral side of the table and used throughout the case to guide external fixation pin positioning. The deformity of the knee joint was fixed using knee joint-spanning circular hexapod external fixation using a four-ring construct [14–16]. Every two rings were positioned orthogonal to the mechanical axis of both the femoral and tibial segments. Every femoral ring and tibial ring was fixed using multiple wires and one or two half-pins as dictated by bone quality. The left lower limb was not weight-bearing for a long period, and the bone was osteoporotic. To avoid straight thin wire cutout, multiple wires were fixed rigidly. The straight thin wires were inserted in the thick parts of the cortical bone as accurately as possible to prevent cutting of the osteoporotic bone [19].

The deformity of knee joint was gradually reduced postoperatively, fixed with 6 telescopic struts, and residual correction was performed postoperatively to achieve desired alignment using the circular hexapod external fixation software. At a pace of 1 mm daily, the tibia was pulled down by 50 mm, the anterior translation of 20 mm and 30° varus deformity was progressively corrected. The joint space was widened, and the alignment was corrected. The nerves, blood vessels, and soft tissues, including those around the knee were extended gradually. Postoperative long bilateral legs standing radiographs were obtained to assess mechanical alignment. Adequate correction was obtained without cut-out of osteoporotic bone for 4 weeks.

#### 3rd stage: hinged TKA

We used a 3D-printed bone model to increase accuracy in the correction of the complex deformity [14–16]. Radiographs and computed tomography (CT) scans revealed a complex deformity with varus, posterior knee dislocation and internal torsion of the left knee. The pre-surgical planning of TKA included quantification of the angular deformity, followed by computer simulated correction, and finally a rehearsal surgery using a 3D printed bone model of modular rotating hinge component of TKA [20].

**Fig. 7:**
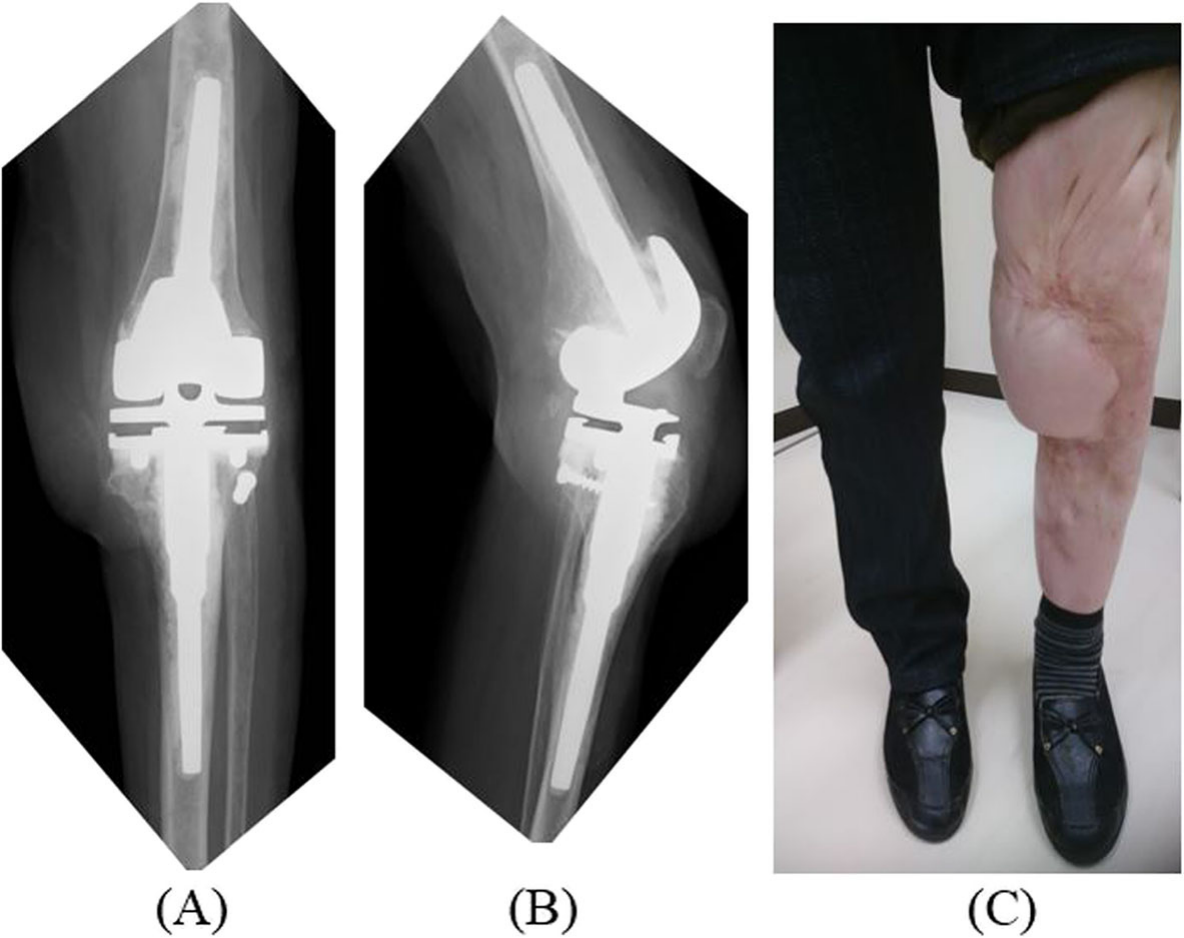
Postoperative X-ray and clinical photograph. **a** Six years postoperative antero-posterior X-ray. **b** Six years postoperative medio-lateral X-ray. **c** Six years postoperative clinical photograph

We performed hinge component of TKA using a medial para-patellar approach. Filling of bone defects was carried out using cement. Postoperative radiographs revealed successful correction of the deformity. Eight-week, 12-week, and 6-year postoperative follow-up examinations showed resolution of her lameness and knee pain. The patient obtained significant improvements in functional outcomes. The Knee Society Score (KSS) increased from 19 to 71/100. At the most recent follow-up, 6 years after TKA, she was fully weight-bearing without walking aids and had no knee pain. The patient had no progressive radiolucent lines with loosening on X-ray (Fig. 7). She was treated with teriparatide for 2 years after surgery and was taking denosumab for osteoporosis at the time of this report.

## Discussion

Medial gastrocnemius flaps occasionally are used to provide soft tissue coverage most commonly [11]. The general indication for using a gastrocnemius flap in this setting was deficient soft tissue over the anterior knee. Gastrocnemius flaps have been used to address difficult soft tissue defects as in this case, in the presence of large burn scar that extended from the proximal medial to distal anterior side of the knee [11]. A popliteo-posterior thigh fasciocutaneous island flap to repair skin defects around the knee is demonstrated the advantages of the method. In a situation in which free tissue transfer is not feasible because of comorbidities, local muscle flaps may be considered. In gastrocnemius flap reconstruction of knee and mid-tibial defects, El-Shazly and Kamal [10, 12, 13] described several useful modifications to increase the rotational arc and dimensions of the medial and lateral heads of the gastrocnemius for coverage of larger wounds.

There is a useful method involving distally-based fasciocutaneous flaps of the posterior surface of the thigh. This leaf-shaped flap extends from the popliteal region to the gluteal sulcus. It possesses an axial arterial network derived from the popliteal artery, reinforced by the perforating arteries derived from the vessel or the profunda femoris artery. The venous return is ensured by venae comitantes and the communicating saphenous vein. Microsurgery to the anterior aspect of the knee is challenging and requires a high degree of skill because the vessel is posterior. We believed the gastrocnemius flap alone would not cover a large wound. Therefore, we combined the popliteo-posterior thigh fasciocutaneous island flap and gastrocnemius flap to cover the wide soft tissue defect around the knee joint. This was suitable for reconstruction of the knee region in the context of her comorbid chronic heart failure. Soft tissue reconstruction, leg length corrections, and TKA are feasible without advanced microsurgery techniques. A soft tissue cover is very important for definitive treatment because it improves the local circulation up to the quality of the mechanical cover, and for bone protection against periprosthetic joint infection [21].

This case is rare and has some limitations that need to be addressed. Firstly, knee joint-bridging circular hexapod external fixation carries a risk of pin-tract infections. When treating severe knee dislocation with a joint-bridging circular hexapod external fixation before TKA, meticulous pin care and immediate treatment with antibiotics are necessary at the first sign of infection [19, 22–28]. Falzarano et al. reported that ESR and CRP levels have a greater diagnostic accuracy in predicting late chronic and early postoperative infections in THA [29]. These markers are valuable supports for the surgeon in monitoring early postoperative superficial pin-tract infection of the circular external fixator, in periprosthetic fractures around the knee. We conducted a postoperative evaluation for the prevention of pin-tract infection using ESR and CRP levels as inflammatory markers [29]. Secondly, the patient may find the use of a circular hexapod external fixation uncomfortable. However, immediate full weight-bearing after surgery is possible because of rigid fixation. Circular hexapod external fixation has great stability as external fixation around the knee joint [30]. The Ilizarov technique is an effective treatment method for complex limb injuries such as road traffic accidents, sports injuries, war trauma, and severe burns around the knee [5–7].

Nowadays the extraordinary improvement of prosthetic implants allows better treatment of severe knee injury [31]. Additionally, to avoid complications related to the treatment of severe contractures and deformities around the knee, one option is to use a circular hexapod external fixation [14–16]. The system works on the basis of the theory of projected geometry, and the mechanical basis of the Stewart-Gough platform. The fixator allows simultaneous correction of multiplanar deformities. Six deformity parameters may be corrected gradually and simultaneously: both angulation and translation in the coronal, sagittal, and axial planes. The minimally invasive nature of the circular fixation, using wires and half-pins to achieve stable fixation in osteoporotic bone, makes this method particularly suited to the treatment of difficult complex deformities of the knee joint, and those with overlying soft tissue problems [14, 15]. The ability to gradually correct the deformity allows the surgeon to apply fixation percutaneously and to avoid opening up the thin and atrophic soft tissue of the burn scar. Any residual deformity is corrected with a gradual correction using the circular hexapod external fixation. Falzarano et al. reported that the occurrence of a limb deformity is a more important factor in the lower extremities than in the upper extremities. The accuracy of knee alignment is always important for knee prosthesis [32, 33].

A patient-specific 3D printed bone model of complex deformity allowed for a satisfactory correction of the lower leg deformity. Additional benefits of using patient-specific 3D printed bone models is reduced surgical time. Implants with specific tibiofemoral junctions are used in cases with non-repairable ligament weakness or in cases with poor bone quality that do not allow for normal knee function [20]. In primary surgery [34], this type of implant is not suitable for standard knee osteoarthritis patients. Its place is in managing large deformities like this case. Cemented stems are used in case of local osteoporosis. Management of this patient was challenging because of her fragile general condition and poor bone strength.

## Conclusions

It is a useful method a combination of soft tissue reconstruction, circular hexapod external fixation and rehearsal surgery using a 3D printed bone model of modular rotating hinge component of TKA to treat a severe knee dislocation due to burn scarring. It was an effective staged surgery that maintained leg length and achieved satisfactory alignment for a severe complex deformity and soft tissue injury caused by a burn injury.

## Data Availability

The datasets used and/or analyzed during the current study are available from the corresponding author on reasonable request.

## Abbreviations

TKA: Total knee arthroplasty
CT: Computed tomography
KSS: The Knee Society Score
